# Incoherent collective cell chemotaxis underlies organ dysmorphia in a model of branchio-oto-renal syndrome

**DOI:** 10.17912/micropub.biology.001342

**Published:** 2024-09-23

**Authors:** Augusto Borges, Filipe Pinto-Teixeira, Indra Wibowo, Hans-Martin Pogoda, Matthias Hammerschmidt, Koichi Kawakami, Hernán López-Schier, Jerónimo Roberto Miranda-Rodríguez

**Affiliations:** 1 Graduate School of Quantitative Biosciences, Ludwig-Maximilians-Universität München, Munich, Germany; 2 Center for Developmental Biology, Université Toulouse III - Paul Sabatier, Toulouse, France; 3 School of Life Sciences and Technology, Bandung Institute of Technology, Bandung, West Java, Indonesia; 4 Institute for Developmental Biology, University of Cologne, Cologne, Germany; 5 Laboratory of Molecular and Developmental Biology, National Institute of Genetics, Mishima, Shizuoka, Japan; 6 Science Division, New York University Abu Dhabi, Saadiyat Island, United Arab Emirates; 7 Department of Neurophysiology and Developmental Neurobiology,, Instituto de Neurobiología, Universidad Nacional Autónoma de México, Juriquilla, México

## Abstract

Mutations in *eya1* cause branchio-oto-renal syndrome (BOR) in humans and the equivalent condition in animal models. BOR is characterized by multi-organ malformations. To better understand the role of Eya1 in organogenesis we used the zebrafish posterior lateral-line primordium. This multicellular tissue moves from head-to-tail at a constant velocity via the simultaneous action of two chemokine receptors, Cxcr4b and Ackr3b (formerly cxcr7b). We found that loss of *eya1* strongly reduces the expression of *ackr3b* , disrupting the coherent motion of the primordium and leading to lateral-line truncations. These findings point to abnormal collective cell chemotaxis as the origin of organ dysmorphia in BOR.

**Figure 1. Loss of eya1 disrupts lateral-line development:**
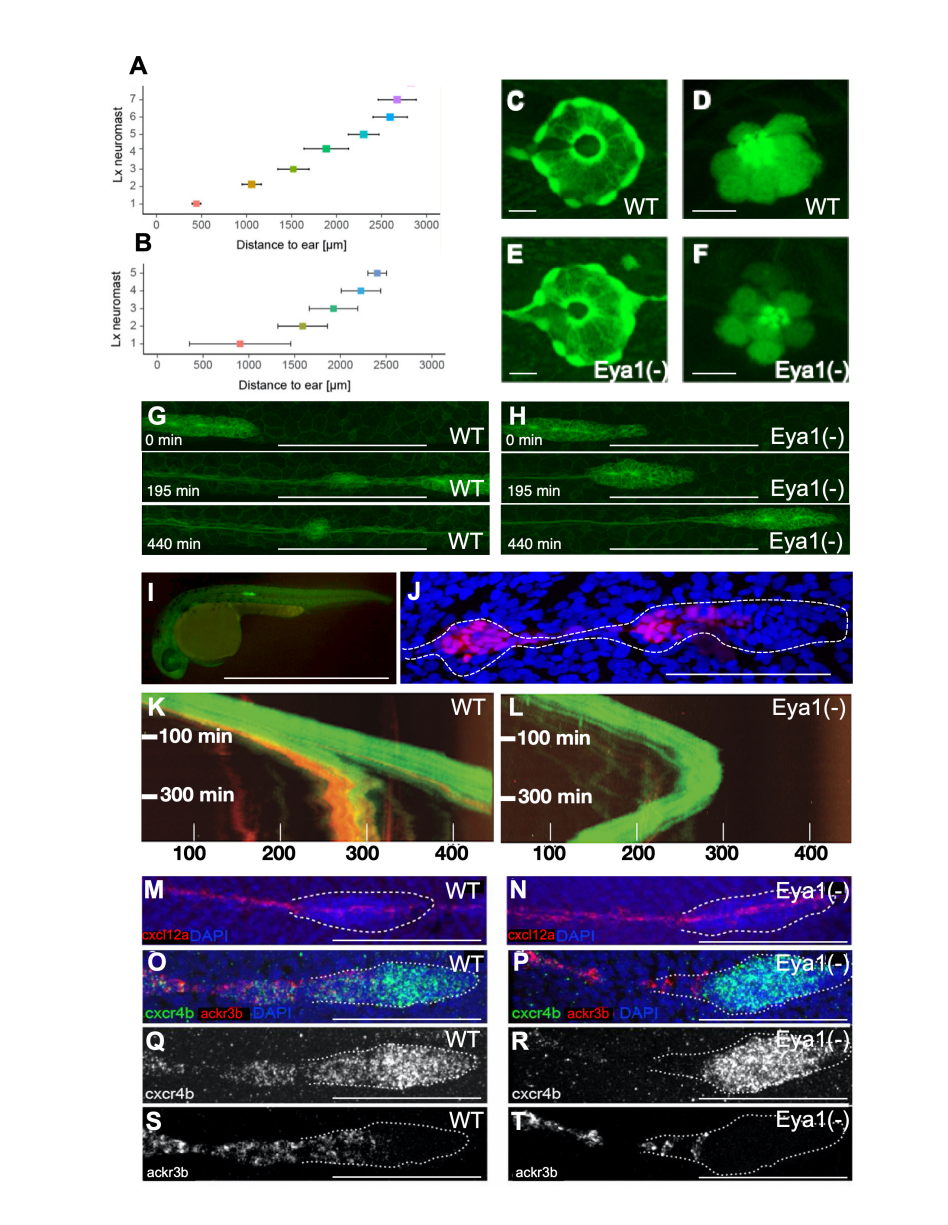
**(A,B)** Plot of the distance (in μm) between the caudal limit of the otic vesicle and the average number of deposited neuromasts in wild-type (E) and *eya1* -mutant (F) specimens at 3 dpf (mean ± s.d.). N= 4 for wild type and N=9 for *eya1* -/-. **(C-F)** Live images of neuromasts of the transgenic lines Et(krt4:EGFP)sqet20 ***(C,E)*** and Et(krt4:EGFP)sqet4 ***(D,F)*** at 6dpf in wild-type (C,D), *eya1* mutants (E,F) revealing supporting cells (C,E) and hair cells (D,F). **(G,H)** High magnification confocal images of a wild type (G) and mutant (H) primordium. At 195 minutes of migration, the wild-type primordium has deposited one pro-neuromast, whereas the *eya1* (-) primordium failed to do so even after 440 minutes. **(I)** A transgenic gSAG181A larva at 30hpf. This line specifically expresses EGFP in the posterior lateral line primordium. **(J)** The primordium and a pro-neuromast in a SAGFF(LF)19A;UAS:RFP larva. SAGFF(LF)19A expresses a Gal4 protein in rear primordial cells, pro-neuromasts and inter-neuromast cells. **(K)** Kymograph from a time-lapse movie focusing on the migrating primordium. The trailing RFP signal driven by SAGFF(LF)19A signal is associated with pro-neuromast rosettogenesis. A few cells expressing 19A:RFP remain in the trailing edge after pro-neuromast deposition. The leading edge advances linearly with a velocity of roughly 80 microns per hour at 28°C. **(L)** Kymograph from a representative *eya1* Crispant larva in the 181A;19A:RFP transgenic background. No neuromast deposition is seen for the duration of the movie. The fish was confirmed to express 19A:RFP in older neuromasts but barely any red fluorescence is seen in the trailing edge of the primordium. The curved shape of the trajectory indicates that after stalling, the primordium performs a U-turn and starts backward migration. Black gaps in the solid green band reflect transient splitting events of the primordium. Units on the x-axis are micrometers (μm). **(M-T)** Representative fluorescent whole-mount *in situ* hybridizations **(M,N)** of *cxcl12a* (red), counterstained with DAPI (blue) to reveal the nuclei for better identification of the primordium (white dotted outline). It shows that the *cxcl12a* gene is expressed along the horizontal myoseptum in the wild type (M) and *eya1* mutants (N). **(O-T)** *cxcr4b* (green) and *ackr3b* (red) gene-expression profiles in wild type (O,Q,S) and *eya1* mutants (P,R,T) The *cxcr4b* gene is strongly expressed in the leading region of primordium in both in wild-type and *eya1* mutants (O-R). The expression of *ackr3b* , however, is strong in the trailing region of the wild-type primordium (overlapping with *cxcr4b* ) (O,Q,S), but almost completely lost in *eya1* mutants, as it is restricted to the very end of the trailing region and never overlaps with *cxcr4b* (P,R,T). HCR conducted in al least 10 samples. Scale bars: C-F 10 μm; G-H 100 μm; I 1mm; J, 100 μm; M-T 100 μm.

## Description

**DESCRIPTION**

The coordinated action of multiple cells governs the development of tissue shape and pattern. Consequently, mutations in genes driving collective cell behavior have profoundly deleterious effects on organogenesis. One gene of particular interest is Eya1, whose loss in vertebrates disrupts the formation of several organs, including the kidney, inner ear, and the lateral line (Kozlowski et al., 2005; Sahly et al., 1999; Seleit et al., 2017; Almasoudi and Schlosser, 2021) . In humans, mutations in Eya1 segregate with 40% of cases of Branchio-Oto-Renal syndrome (BOR) (Abdelhak et al., 1997; Sánchez-Valle et al., 2010; Krug et al., 2011). Standard treatments for BOR over the past 25 years have been kidney transplants, dialysis and hearing aids (Smith, 1993; Tian et al., 2022) . More innovative interventions are lacking in part because the cellular mechanisms that are disrupted in BOR remain obscure (Soni et al., 2021) . Here we combine forward- and reverse-genetic analyses with live imaging to study a model of BOR in zebrafish.

Alterations of the Cxcl12a (formerly Sdf1a) chemokine receptors CXCR4b and Ackr3b (formely CXCR7b) lead to defects in neuromast deposition during the formation of the lateral line (Venkiteswaran et al. 2013; Donà et al., 2013). Therefore, we speculated that mutations affecting the number of neuromasts will identify factors involved in chemokine signaling. Following this rationale, we analyzed zebrafish carrying a loss-of-function mutation in *eya1* (Kozlowski et al., 2005; Nica et al., 2006) . Using somatic CRISPR/Cas9-mediated genome engineering we mutated *eya1* and the fluorescent enhancer-trap line SqET20 to mark non-sensory supporting cells, and SqET4 to highlight the mechanosensory hair cells in neuromasts (Parinov et al., 2004) . We found that mutants produce fewer neuromasts within a truncated lateral line ( Fig. 1A -B). However, the survival of mutant neuromasts over the course of 4 days after their deposition was not affected by the loss of *eya1* ( Fig. 1C -F). These data indicate that the lateral-line defects in *eya1* mutants arise during development and not from post-embryonic degeneration of neuromasts. When looking at early embryos, we found that loss of *eya1* delayed the migration of primordium ( Fig. 1G -H).

Next, we focused on primordium dynamics from the onset of migration by *in toto* videomicroscopy, combining two fluorescent enhancer-trap lines, Tg[gSAG181A] and Tg[SAGFF(LF)19A] (Kawakami et al., 2004) . Tg[gSAG181A] is unique in that it is the only known line that expresses EGFP exclusively in the posterior lateral-line primordium ( Fig. 1I ). We found that Tg[gSAG181A] is an insertion near the SAM and SH3 domain containing 1a ( *sash1a* ) *locus* on chromosome 20. The Tg[SAGFF(LF)19A] is an insertion of a Gal4 transgene into *ebf3* *locus* (Kuriki et al., 2020) . When combined with a UAS-driven RFP, it drives expression in the rear part of the primordium and in the deposited neuromasts ( Fig. 1J ). The combined Tg[gSAG181A;SAGFF(LF)19A;UAS:RFP] showed that wild-type primordia move at a constant velocity of around 80 μm/hour ( Fig. 1K and Supp. Movie 1), whereas *eya1* -deficient primordia undergo cycles of migration and stalling, averaging a markedly reduced speed of 14 μm/hour (Fig 1L). Moreover, primordia lacking *eya1* sometimes make U-turns to move back towards the head ( Fig. 1L and Supp. Movie 2). Therefore, the loss of *eya1* does not block primordium migration, but instead creates pronounced defects in its otherwise coherent motion. The expression of *cxcl12a* chemokine along the migratory path, and the chemokine receptor *cxcr4b* in the front of the primordium remained normal in *eya1* -mutant fish ( Fig. 1M -R). By contrast, the expression of *ackr3b* in the trailing part of the primordium was strongly diminished ( Fig. 1O -P,S-T). Therefore, loss of *eya1* disrupts primordium migration due to reduced expression of *ackr3b* , which in turn may saturate the CXCR4b receptor by maintaining abnormally high levels of Cxcl12a in the trailing part of the primordium.

Based on our findings, we propose that the Eya1 protein may also govern coherent collective cell movement during otic and renal development mainly via chemokine signaling. Therefore, our results shed new light on the role of Eya1 on collective cell migration and suggest potential avenues to explore novel therapeutic strategies for human patients. Given that the causative mutation for over 50% of BOR cases is not yet known, we predict that the lateral line of zebrafish will remain a powerful model to validate genomic polymorphisms from GWAS studies of BOR patients and generate novel cellular and molecular insights with translational potential. On this regard, our findings raise the possibility that augmenting residual CXCR7 protein activity may improve the outcome of *eya1* mutations in humans (Jiang et al., 2021; Hughes and Nibbs, 2018) . It also encourages the development of tissue engineering approaches to control collective cell migration aimed at clinical applications (Manivannan et al., 2012) .

**CONFLICT OF INTEREST STATEMENT**

HL-S is scientific advisor and paid consultant for Sensorion (France). The company had no role in this study. No conflict of interests exists.

## Methods

MATERIALS AND METHODS

Zebrafish animals and strains

Fish used were maintained under standardized conditions. Experiments were performed in accordance with protocols approved by the Ethical Committee of Animal Experimentation of the Helmholtz Zentrum München, the German Animal Welfare act Tierschutzgesetz §11, Abs. 1, Nr. 1, Haltungserlaubnis according to the European Union animal welfare, and under protocol number Gz.:55.2-1-54-2532-202-2014 and Gz.:55.2-2532.Vet_02-17-187 from the “Regierung von Oberbayern” (Germany). Eggs were collected from natural spawning and maintained at 28.5°C. Embryos were staged by hours post fertilization (hpf). The *eya* mutant allele used in this study is *eya1^tm90b* (Nica et al., 2006) . Embryos were genotyped according to Kozlowski et al. (Kozlowski et al., 2005) . Transgenic lines used were Et(krt4:EGFP)sqet4 and Et(krt4:EGFP)sqet20 (Pinto-Teixeira et al., 2015) , Tg[Cldnb:lynEGFP]zf106Tg (Haas and Gilmour, 2006) , gSAG181A ( *Gt(T2KSAG)nkgsag181A)* and SAGFF(LF)19A = Et(T2KSAGFFLF)nkSAGFFLF19AEt (Kawakami et al., 2004) .

Somatic CRISPR gene knock-out

Crispr somatic mutagenesis of *eya1* was done with 4 sgRNAs (Wu et al., 2018) . A 1 mg/ml equimolar mixture of 4 sgRNAs, transcribed with MEGAshortscript T7 (Thermo Fischer), 5 mM Cas9 protein (Sigma), and 300 mM KCl, was injected into one-cell stage embryos. The sequences in the eya1 gene targeted by each sgRNA are the following: CTTCCACTTACTCGGCTGTG,TTGTCAATGTTGGGACCGTT, GACGTACCTTCAGTGCCATT, AGAGCCGTCTGCTACAGAGG

Whole-mount *in situ* hybridization (ISH)

For ISH, antisense digoxigenin- and fluorescein-labeled riboprobes were synthesized according to manufacturer’s instructions (Roche) by using T7/SP6/T3 RNA polymerases. Probes used were: cxcl12a, cxcr4b, ackr3b. Whole-mount two-color fluorescence ISH was performed using anti-DIG and -fluorescein POD antibodies (Roche) and Tyramide Signal Amplification (TSA, PerkinElmer) to detect the riboprobes. Briefly, samples were fixed in 4% paraformaldehyde (PFA) for 24h at 4°C, permeabilized with methanol and cooled to 20°C. Next day, samples were rehydrated, treatment with proteinase K and post-fixed in PFA for 20 min at room temperature. The samples were washed with PBST between the steps. Probe hybridization buffer was used for the prehybridization for 30 min at 37°C and the samples were incubated in the probe solution, prepared following the manufacturer’s instructions, overnight at 37°C. After removing the probe solution, washing the samples and incubating them in the pre-amplification buffer, the samples were incubated in the hairpin mixture overnight in the dark at room temperature. Finally, after several washes with SSCT, the cell nuclei were stained with DAPI (40,6-diamidino-2-phenylindole, Sigma) 1 hour at room temperature.

Imaging and time-lapse video microscopy

For whole-mount ISH, embryos were de-yolked, flat mounted and photographed with an Olympus BX61 microscope using 20X or 40X dry objectives with transmission light. Whole embryo images were acquired on a Leica MZ10 stereomicroscope. Fluorescent images were acquired using either a Leica SP5 or SPE microscope using 20X dry objective or 40X oil immersion objective. Images were processed using Imaris and/or ImageJ software packages, and assembled with Adobe Photoshop CS2, Adobe Illustrator CS2, and Macromedia FreeHand MX. For time-lapse imaging, staged and de-chorionated embryos were anesthetized with Tricaine and mounted in 0.8-1% low-melting-point agarose on a glass-bottom culture dish (MatTek) as previously described (Torres-Mejía et al., 2020) . Z-stack series were acquired every 4-10 min using a 20X dry objective of Leica SPE or SP5 confocal microscope. All movies were processed with the Imaris or ImageJ software packages. An unpaired two-tailed T test with Welch’s correction was used to compare the position of neuromast L4 in *eya1* mutants and wild-type siblings. Statistics were performed using the GraphPad Prism software and Excel running QI Macros.

## Extended Data

Description: Movie 1. Resource Type: Audiovisual. DOI: 10.22002/h880t-ea128

Description: Movie 2. Resource Type: Audiovisual. DOI: 10.22002/d6d9v-09443

## References

[R1] Abdelhak S., Kalatzis V., Heilig R., Compain S., Samson D., Vincent C., et al., Leibovici M.. 1997. A human homologue of the Drosophila eyes absent gene underlies Branchio-Oto-Renal (BOR) syndrome and identifies a novel gene family. Nat. Genet. 15: 157. 10.1038/ng0297-157 9020840

[R2] Almasoudi S.H., Schlosser G.. 2021. Otic Neurogenesis in Xenopus laevis: Proliferation, Differentiation, and the Role of Eya1. Front. Neuroanat. 15: 722374. 10.3389/fnana.2021.722374 PMC8488300 34616280

[R3] Donà E., Barry J.D., Valentin G., Quirin C., Khmelinskii A., Kunze A., et al., Huber W.. 2013. Directional tissue migration through a self-generated chemokine gradient. Nature. 503: 285. 10.1038/nature12635 24067609

[R4] Haas P., Gilmour D.. 2006. Chemokine signaling mediates self-organizing tissue migration in the zebrafish lateral line. Dev. Cell. 10: 673. 10.1016/j.devcel.2006.02.019 16678780

[R5] Hughes C.E., Nibbs R.J.B.. 2018. A guide to chemokines and their receptors. FEBS J. 285: 2944. 10.1111/febs.14466 PMC6120486 29637711

[R6] Jiang C., Li R., Xiu C., Ma X., Hu H., Wei L., et al., Zhao J.. 2021. Upregulating CXCR7 accelerates endothelial progenitor cell-mediated endothelial repair by activating Akt/Keap-1/Nrf2 signaling in diabetes mellitus. Stem Cell Res. Ther. 12: 264. 10.1186/s13287-021-02324-7 PMC8091720 33941256

[R7] Kawakami K., Takeda H., Kawakami N., Kobayashi M., Matsuda N., Mishina M.. 2004. A Transposon-Mediated Gene Trap Approach Identifies Developmentally Regulated Genes in Zebrafish. Dev. Cell. 7: 133. 10.1016/j.devcel.2004.06.005 15239961

[R8] Kozlowski D.J., Whitfield T.T., Hukriede N.A., Lam W.K., Weinberg E.S.. 2005. The zebrafish dog-eared mutation disrupts eya1, a gene required for cell survival and differentiation in the inner ear and lateral line. Dev. Biol. 277: 27. 10.1016/j.ydbio.2004.08.033 15572137

[R9] Krug P., Morinière V., Marlin S., Koubi V., Gabriel H.D., Colin E., et al., Heidet L.. 2011. Mutation screening of the EYA1, SIX1, and SIX5 genes in a large cohort of patients harboring branchio-oto-renal syndrome calls into question the pathogenic role of SIX5 mutations. Hum. Mutat. 32: 183. 10.1002/humu.21402 21280147

[R10] Kuriki M., Sato F., Arai H.N., Sogabe M., Kaneko M., Kiyonari Kawakami, et al., Sehara-Fujisawa A.. 2020. Transient and lineage-restricted requirement of Ebf3 for sternum ossification. Development. 147: 186239. 10.1242/dev.186239 PMC7240299 32398354

[R11] Manivannan S., Gleghorn J.P., Nelson C.M.. 2012. Engineered Tissues to Quantify Collective Cell Migration During Morphogenesis. 10.1007/978-1-61779-851-1_16 22639261

[R12] Nica G., Herzog W., Sonntag C., Nowak M., Schwarz H., Zapata A.G., Hammerschmidt M.. 2006. Eya1 is required for lineage-specific differentiation, but not for cell survival in the zebrafish adenohypophysis. Dev. Biol. 292: 189. 10.1016/j.ydbio.2005.12.036 16458879

[R13] Parinov S., Kondrichin I., Korzh V., Emelyanov A.. 2004. Tol2 transposon-mediated enhancer trap to identify developmentally regulated zebrafish genes in vivo. Dev. Dyn. 231: 449. 10.1002/dvdy.20157 15366023

[R14] Pinto-Teixeira F., Viader-Llargués O., Torres-Mejía E., Turan M., González-Gualda E., Pola-Morell L., López-Schier H.. 2015. Inexhaustible hair-cell regeneration in young and aged zebrafish. Biol. Open. 4: 903. 10.1242/bio.012112 PMC4571094 26002931

[R15] Sahly I., Andermann P., Petit C.. 1999. The zebrafish eya1 gene and its expression pattern during embryogenesis. Development Genes and Evolution. 209: 399. 10.1007/s004270050270 10370123

[R16] Sanchez-Valle A., Wang X., Potocki L., Xia Z., Kang S.-H.L., Carlin M.E., et al., Brundage E.K.. 2010. HERV-mediated genomic rearrangement of EYA1 in an individual with branchio-oto-renal syndrome. Am. J. Med. Genet. 152A: 2854. 10.1002/ajmg.a.33686 PMC3605882 20979191

[R17] Schumacher L.. 2019. Collective Cell Migration in Development. Cell Migrations: Causes and Functions: 105. 10.1007/978-3-030-17593-1_7 31612456

[R18] Seleit A., Krämer I., Ambrosio E., Dross N., Engel U., Centanin L.. 2017. Sequential organogenesis sets two parallel sensory lines in medaka. Development. dev.142752 10.1242/dev.142752 PMC5312036 28087632

[R19] Smith R.J., Adam M.P.), Mirzaa G.M.), Pagon R.A.), Wallace S.E.), Bean L.J.), Gripp K.W.), Amemiya A.. 1993. Branchiootorenal Spectrum Disorder. 20301554

[R20] Soni U.K., Roychoudhury K., Hegde R.S.. 2021. The Eyes Absent proteins in development and in developmental disorders. Biochem. Soc. Trans. 49: 1397. 10.1042/BST20201302 PMC8286820 34196366

[R21] Tian L., West N., Cayé-Thomasen P.. 2022. Cochlear implantation in Branchiootorenal syndrome – case report and review of the literature. Coch. Imp. Internat. 23: 52. 10.1080/14670100.2021.1973209 34498539

[R22] Torres-Mejía E., Trümbach D., Kleeberger C., Dornseifer U., Orschmann T., Bäcker T., et al., Desbordes S.C.. 2020. Sox2 controls Schwann cell self-organization through fibronectin fibrillogenesis. Sci Rep. 10 10.1038/s41598-019-56877-y PMC7005302 32029747

[R23] Vasilyev A., Liu Y., Mudumana S., Mangos S., Lam P.-Y., Majumdar A., et al., Korzh V.. 2009. Collective Cell Migration Drives Morphogenesis of the Kidney Nephron. PLoS Biol. 7: 1000009. 10.1371/journal.pbio.1000009 PMC2613420 19127979

[R24] Venkiteswaran G., Lewellis S.W., Wang J., Reynolds E., Nicholson C., Knaut H.. 2013. Generation and Dynamics of an Endogenous, Self-Generated Signaling Gradient across a Migrating Tissue. Cell. 155: 674. 10.1016/j.cell.2013.09.046 PMC3842034 24119842

[R25] Wu, RS., Lam II, Clay H, Duong DN , Deo RC , Coughlin SR. (2018). A Rapid Method for directed Gene Knockout for Screening in G0 Zebrafish. *Dev. Cell* 46(1):112-125.e4.. 2018. A Rapid Method for. 10.1016/j.devcel.2018.06.003 29974860

